# Apatinib combined with temozolomide in diffuse midline glioma: a novel and effective therapy

**DOI:** 10.1186/s12885-024-12373-9

**Published:** 2024-06-21

**Authors:** Yu-An Li, Chuan Zhao, Jing-Jing Ge, Cheng Li, Feng-Jun Xue, Shao-Pei Qi, Chi Zhao, Chen-Chen Kong, Jun-Ping Zhang

**Affiliations:** Department of Neuro-Oncology, Sanbo Brain Hospital, Capital Medical University, Beijing, China

**Keywords:** Diffuse midline glioma, H3 K27M-mutant, Apatinib, Temozolomide, Chemotherapy

## Abstract

**Purpose:**

Diffuse midline glioma (DMG), H3 K27M-mutant is a type of diffuse high-grade glioma that occurs in the brain midline carrying an extremely poor prognosis under the best efforts of surgery, radiation, and other therapies. For better therapy, we explored the efficacy and toxicity of a novel therapy that combines apatinib and temozolomide in DMG.

**Methods:**

A retrospective analysis of 32 patients with DMG who underwent apatinib plus temozolomide treatment was performed. Apatinib was given 500 mg in adults, 250 mg in pediatric patients once daily. Temozolomide was administered at 200 mg/m^2^/d according to the standard 5/28 days regimen. The main clinical data included basic information of patients, radiological and pathological characteristics of tumors, treatment, adverse reactions, prognosis.

**Results:**

The objective response rate was 24.1%, and the disease control rate was 79.3%. The median PFS of all patients was 5.8 months, and median OS was 10.3 months. A total of 236 cycles of treatment were available for safety assessment and the toxicity of the combination therapy was relatively well tolerated. The most common grade 3 toxicities were myelosuppression including leukopenia (5.08%), neutropenia (4.24%), lymphopenia (2.12%), thrombocytopenia (1.69%) and anemia (1.27%). Grade 4 toxicities included neutropenia (2.12%), thrombocytopenia (2.12%) and proteinuria (1.69%). All the adverse events were relieved after symptomatic treatment or dose reduction.

**Conclusions:**

Apatinib plus temozolomide could be an effective regimen with manageable toxicities and favorable efficacy and may outperform temozolomide monotherapy, particularly in newly diagnosed adults with tumors located outside the pons. The novel therapy deserves further investigation in adult DMG patients.

**Supplementary Information:**

The online version contains supplementary material available at 10.1186/s12885-024-12373-9.

## Key points

1. The novel therapy that combines apatinib and temozolomide could be an effective regimen with manageable toxicities in DMGs.

2. Apatinib plus temozolomide is a therapeutic improved with favorable efficacy and at least superior to temozolomide monotherapy.

3. It was confirmed that some adult and thalamic subgroup patients with DMG could benefit from the application of apatinib plus temozolomide.

**Supplementary Information:**

The online version contains supplementary material available at 10.1186/s12885-024-12373-9.

## Importance of the study

This study identified that apatinib plus temozolomide could be an effective regimen with manageable toxicities in DMGs. Overall progression-free survival was associated with age of onset, the best response, and recurrence. Overall survival was associated with age of onset, the best response, and tumor recurrence. Our treatment regimen was an effective trial for this type of high-grade malignancy that is tricky to treat, and the results of the study were at least superior to those of most competing studies. Our study identified that adult, thalamic subgroups are likely to be clinically beneficial. Interestingly, we found that recurrence occurred about half a year after stopping treatment, suggesting the need for long-term anti-tumor therapy.

## Introduction

Diffuse midline glioma (DMG), H3 K27M-mutant is a type of diffuse high-grade glioma that occurs in the midline, which was first recognized in the 2016 World Health Organization (WHO) Classification of Tumors of the Central Nervous System (CNS) and revised as “Diffuse midline glioma, H3 K27-altered” in the 2021’s latest edition [1]. The characteristic molecular pathological change is the H3 K27 methylation level comprehensive decreasing caused by the substitution of lysine at position 27 by methionine in histone 3.3 or histone 3.1 coding gene H3F3A or HIST1H3B/C or other epigenetic events [2], more than 80% of diffuse intrinsic pontine gliomas (DIPGs) in the previous classification can be classified as DMG [3]. DMG progresses rapidly, is difficult to treat, and has a poor prognosis. It’s median overall survival is about 11 months [4, 5]. The current standard radiation therapy can only prolong the survival by about 3 months [6]. Therefore, experimental targeted therapy after radiotherapy is currently the main systemic treatment for it [7].

Previous researches suggested that microvascular proliferation and vascular permeability changes are important events in high-grade glioma, and growth factor receptor pathway changes are the molecular pathological basis, so anti-angiogenic targeted therapy has unique potential. Vascular endothelial growth factor (VEGF) is one of the most important therapeutic targets. In the VEGF family, vascular endothelial growth factor receptor-2 (VEGFR-2) is a key receptor regulating angiogenesis [8].

Apatinib is an oral small-molecule VEGFR-2-targeted inhibitor that prevents activation of downstream signaling pathways by inhibiting VEGFR-2 phosphorylation [9]. Based on the results of phase III clinical trials, apatinib has been approved in China for patients with advanced gastric cancer [10]. In addition, apatinib has also been shown in other clinical trials to improve the prognosis of other advanced solid tumors, such as ovarian cancer, hepatocellular carcinoma, and advanced non-small cell lung cancer [11–13]. In nervous system tumors, preclinical studies have confirmed that apatinib and temozolomide have a synergistic effect [9]; clinical trials and a study of our team have shown that combination of apatinib and temozolomide can improve the prognosis of patients with recurrent glioblastoma without affecting the sensitivity of the tumor to other anti-angiogenic drugs [14–18], suggesting that apatinib has therapeutic potential in high-grade glioma.

Based on the above, we reviewed and analyzed the characteristics and treatment process of DMG patients, who were treated with temozolomide plus apatinib in our department and evaluated the efficacy and safety of this treatment regimen in diffuse midline glioma, H3 K27-mutant.

## Materials and methods

### Patients

All patients enrolled in this retrospective study with diffuse midline glioma, H3 K27M mutant, were treated with apatinib plus temozolomide in Sanbo Brain Hospital of Capital Medical University from December 2016 to December 2021. This study was approved by the Ethics Committee of Sanbo Brain Hospital of Capital Medical University. Patients signed an informed consent form prior to treatment, authorizing the use of their personal information for research purposes.

Inclusion criteria were as follows: age ≥ 3 years; baseline Karnovsky performance score (KPS) or Lansky performance score (LPS) ≥ 50; diffuse midline glioma with H3 K27M mutation diagnosed by immunohistochemistry (IHC) or sequencing; completed the standard radiotherapy(total dose 54 Gy, more than 6 weeks, combined/non-combined concurrent chemoradiotherapy) before enrolled; received at least one cycle of treatment with a combination of temozolomide and apatinib; had at least one post-treatment radiological follow-up. Patients with incomplete medical records were excluded.

### Treatment

Adult received oral apatinib 500 mg, and pediatric patients received oral apatinib 250 mg once a day, combined with temozolomide. Temozolomide was administered at a dose of 200 mg/m^2^/d according to a standard 5/28-day regimen. One treatment cycle was defined as 28 days (4 weeks). Patients continued treatment for up to 2 years, or until disease progression or unacceptable toxicity.

The clinical data collected include age, gender, KPS/LPS, time of diagnosis, previous treatment, tumor location, tumor size, pathological type, molecular pathological features, treatment start time, treatment cycle, radiological response, dose change, adverse reactions, date of progression, other treatments since progression, and date of death.

### Assessments

Radiological responses were classified according to Response Assessment in Pediatric Neuro-Oncology (RAPNO) criteria [19]. Contrast-enhanced MRI and fluid attenuated inversion recovery (FLAIR) examinations were performed at baseline and every 2 cycles after starting treatment until disease progression. Diffusion and perfusion-weighted imaging were used to differentiate pseudoprogression from true progression. Toxicity was classified according to the Common Terminology Criteria for Adverse Events (CTCAE) 5.0.

### Statistical analyzes

Observation indicators include median progression-free survival (mPFS), median overall survival (mOS), PFS rate at 1 year (1y-PFS), OS rate at 1 year (1y-OS) and objective response rate (ORR). PFS was defined as the time from the initiation of treatment to disease progression, death from any cause, or last follow-up; OS was defined as the time from initiation of treatment to death from any cause or last follow-up.

SPSS 22.0 software was used for statistical analysis, and R 4.0 software was used for drawing statistical graphs. Categorical variables are described with numbers and percentages, and continuous variables are described with medians and ranges. Survival curves for PFS and OS were analyzed using the Kaplan–Meier method. The relationship between survival and categorical predictors was assessed with the Log-Rank test. *P* value < 0.05 was considered statistically significant.

## Results

### Patient characteristics

Between December 2016 and December 2021, a total of 32 patients were identified, of which 16 were newly diagnosed patients (50%) and 16 were recurrent (defined as disease progression after biopsy/surgery, radiotherapy, and systemic therapy with at least one regimen patients) (50%) (Table 1). Among them, there were 19 males (59.4%) and 13 females (40.6%), with a male to female ratio of 1.46. The median age at diagnosis was 12.96 years old (range: 5.4–55 years old), of which 22 patients (68.8%) were under 18 years old, and 10 patients (31.2%) were adults. Tumors were in the pons in 18 patients (56.3%), in the thalamus in 9 patients (28.1%), in the ventricles in 4 patients (12.5%), and in the spinal cord in 1 case (3.1%). The most common primary lesion location in pediatric patients was the pons (16/22, 72.7%), in adult patients it was the thalamus (5/10, 50%). 7 of the recurrent patients (21.9%) had disseminated disease before treatment. All 32 patients were confirmed to have H3K27M mutation, among which 31 cases (96.9%) had H3.3 mutation and 1 case (3.8%) had H3.1 mutation. 22 cases underwent ATRX IHC or genetic testing, of which 4 cases (18.2%) were found to be ATRX loss, and 18 cases (81.8%) had complete expression. IDH1/2 mutations were investigated in 29 patients, all of which were wild type; MGMT promoter methylation status was assessed in 30 patients, of which MGMT promoter methylation was found in 5 cases (16.7%).

**Table 1:** Characteristics of 32 patients

Characteristics	Number	Group		Frequency (%)
		**Newly diagnosed (%)**	**Recurrent (%)**	
**Gender**				
Male	19	10 (62.5%)	9 (56.3%)	59.4
Female	13	6 (37.5%)	7 (43.7%)	40.6
**Age**				
< 18	22	10 (62.5%)	12 (75%)	68.7
≥ 18	10	6 (37.5%)	4 (25%)	31.3
**Location**				
Pons	18	8 (50%)	10 (62.5%)	56.3
Thalamus	9	5 (31.3%)	4 (25%)	28.1
Ventricles	4	2 (12.5%)	2 (12.5%)	12.5
Spinal cord	1	1 (6.2%)	0	3.1
**Spread**				
No	25	16 (100%)	9 (56.3%)	78.1
Yes	7	0	7 (43.7%)	21.9
**Diameter (cm)**				
≤ 3	7	2 (12.5%)	5 (31.3%)	21.9
> 3	25	14 (87.5%)	11 (68.7%)	78.1
**WHO Grade**				
WHO 2	5	2 (12.5%)	3 (18.7%)	15.6
WHO 3	10	2 (12.5%)	8 (50%)	31.3
WHO 4	17	12 (75%)	5 (31.3%)	53.1
**Surgical intervention**				
Biopsy only	1	1 (6.2%)	0	3.1
Partial resection	24	1 (62.5%)	14 (87.5%)	75
Gross/sub total	7	5 (31.3%)	2 (12.5%)	21.9
**H3 K27 mutant type**				
H3.3 mutant	31	15 (93.8%)	16 (100%)	96.9
H3.1 mutant	1	1 (6.2%)	0	3.1
**ATRX**				
Loss	4	0	4	12.5
Complete	18	13	5	56.3
Unknown	10			31.2
**Ki67 (%)**				
≤ 5	2	2	0	6.3
> 5	24	11	13	75.0
Unknown	6			18.7
**MGMT promoter methylation**				
Methylated	5	3	2	15.6
Unmethylated	25	13	12	78.1
Unknown	2			6.3
**IDH1/2 mutant**				
Mutant	0			0
Wild type	29	15	14	90.6
Unknown	3			9.4
**TERT promoter**				
Mutant	0			0
Wild type	24	14	10	75.0
Unknown	8			25.0

## Response to treatment

**Fig. 1:**
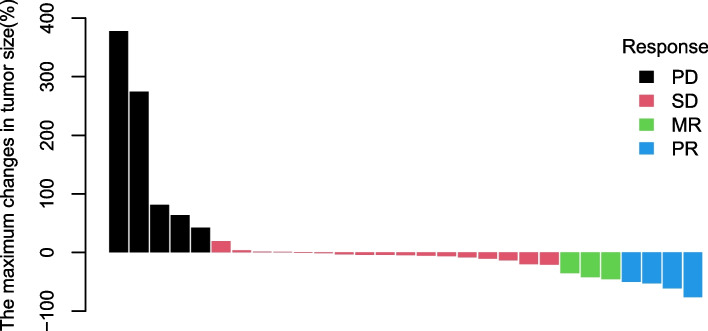
Responses of 29 patients. Three in all 32 patients had no evaluation profile, and the other 29 patients were available for radiological evaluation. The blue bars indicate the 4 patients with DMGs who had a partial response to apatinib plus temozolomide therapy. The red bars indicate the 17 patients having stable disease. The green bars indicate the 3 patients who had a minor response and the black bars indicate that 5 patients had a progressive disease. The horizontal lines indicate the corresponding measures for each type of response. PR: partial response; SD: stable disease; MR: minor response; PD: progressive disease

The response to the combination therapy is shown in Fig. 1. Of all patients, 3 had no measurable disease at baseline evaluation before treatment, and the other 29 patients (13 newly diagnosed patients,16 recurrent patients) were available for radiological evaluation. Among newly diagnosed patients, 2 patients had partial response (PR) (15.4%), 10 patients had stable disease (76.9%), and one patient had progressive disease (0.7%). The objective response rate (ORR) was 15.4%, and the disease control rate was 92.3%.

**Fig. 2:**
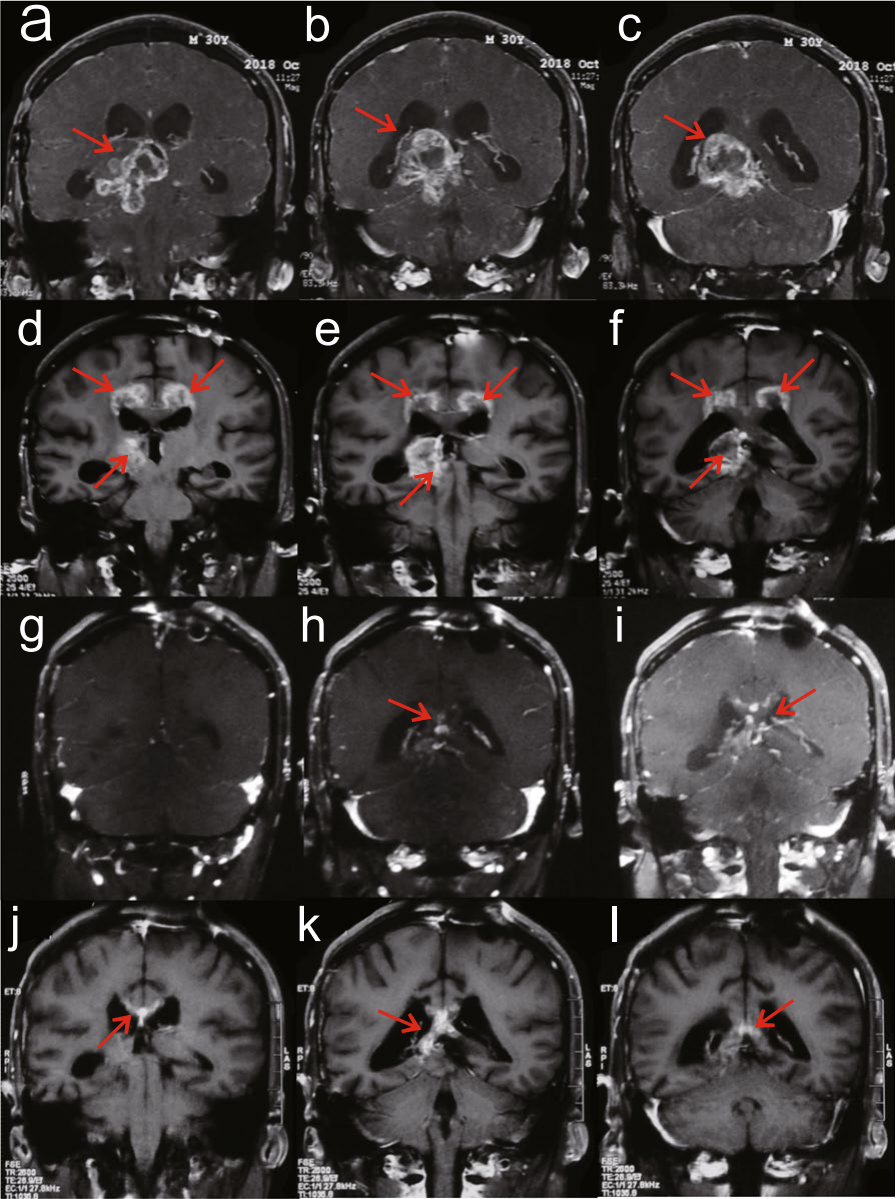
The MRI of one patient who had a partial response. **a-c** before treatment; **d-f** after 10 cycles; **g-i** after 17 cycles, response was PR; **j-l** when the whole treatment for 2 years was completed (red arrows, tumor)

Among recurrent patients, 2 patients had partial response (PR) (12.5%), 3 patients had minor response (18.7%), 16 patients had stable disease (37.5%), and 5 patients (one had no obvious changes in images, but the symptoms were significantly worse) had progressive disease (31.3%). The objective response rate (ORR) was 31.3%, and the disease control rate was 68.8%. Figure 2 shows the MRI of one recurrent patient who got PR. While treatment, the tumor reduced slowly. After 17 cycles of treatment, PR was achieved and lasted for nearly 10 months.

## Survival

**Fig. 3:**
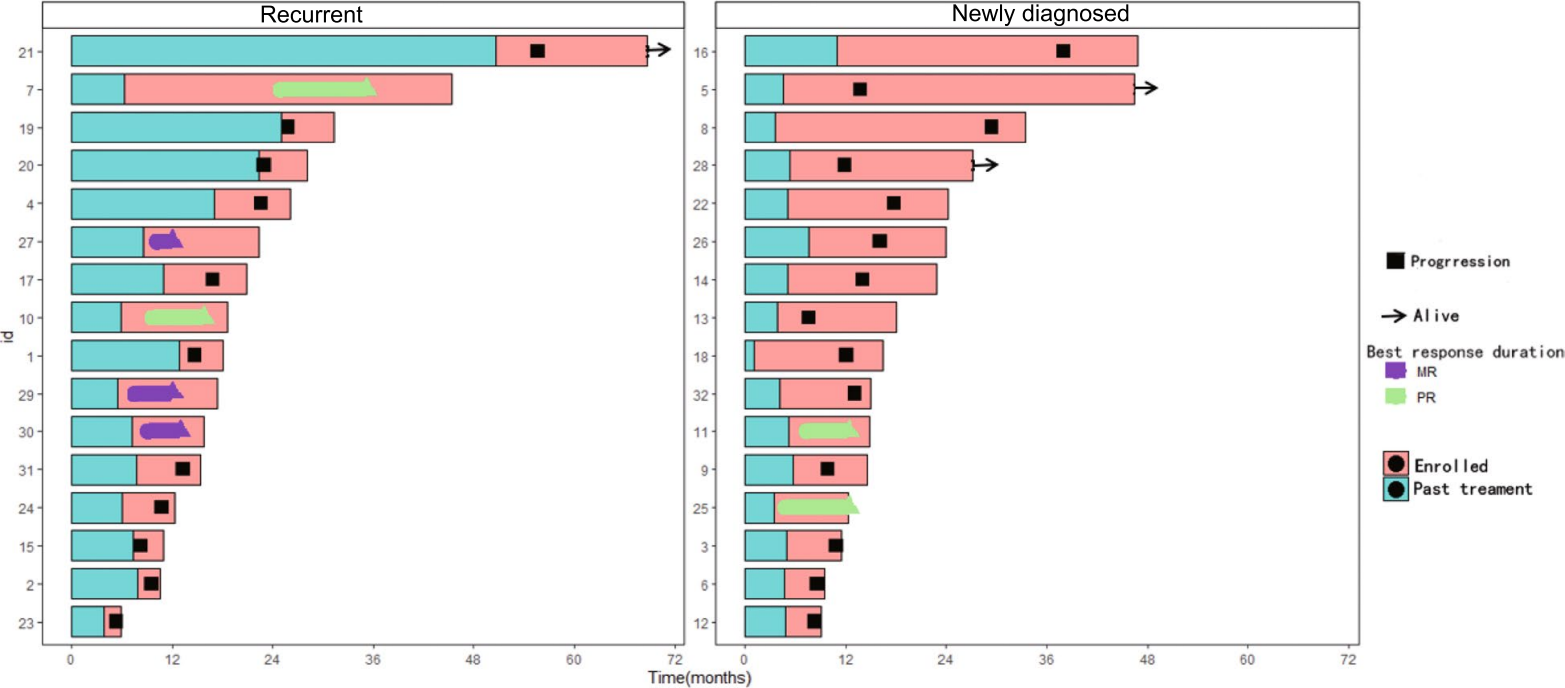
Swimmer plots of treatment history of recurrent and newly diagnosed patients. Progression (black square): tumor progression occurred from the time of study treatment. Alive (black arrow): the patients were still alive at the time of follow-up. The best response duration: MR (the purple bar); PR (the green bar); Enrolled (the light red bar): from the time patients were enrolled in the study. Past treatment (the blue bar): the patient's treatment period prior to this study

Median follow-up time is 9.8 months (range 2.1—42.6 months, last follow-up date: September 1st, 2022). Among all patients, 27 patients experienced disease progression during the treatment of this regimen. Among the other 5 patients, 2 patients had no disease progression at the last follow-up after 9.0 months and 16.1 months after the initiation of treatment due to personal withdrawal respectively; the other 3 patients stopped treatment after receiving the regimen for 2 years, and disease progressed during the follow-up period. The course of all patients is shown in Fig. 3.

**Fig. 4:**
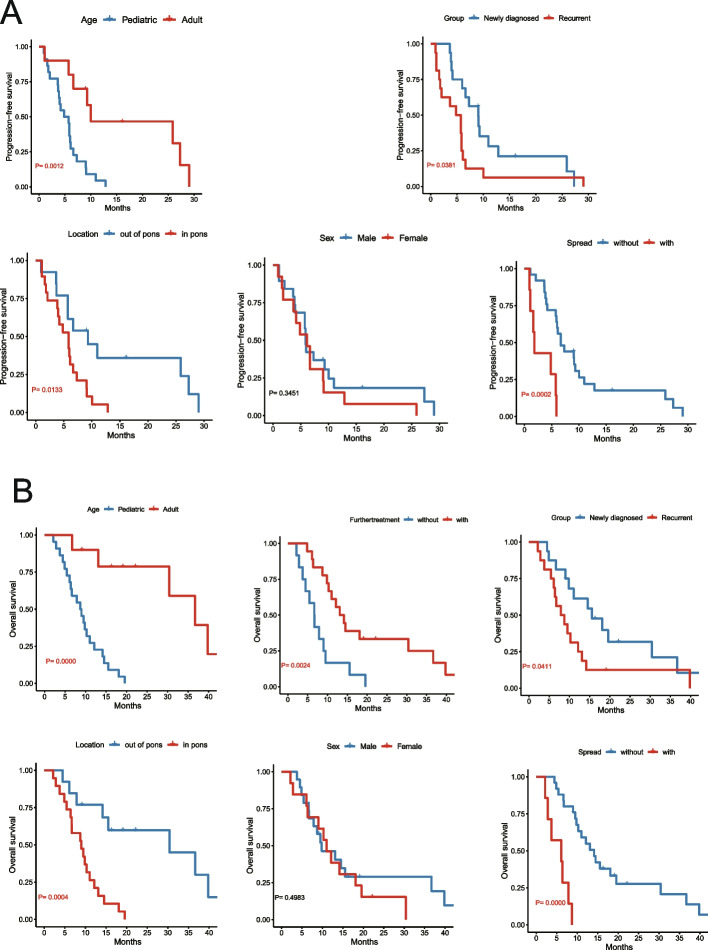
Prognostic analysis of all patients. **a** Progression-free survival was compared between groups based on age, tumor location, recurrence, sex, and spread; **b** Overall survival was compared between groups based on age, tumor location, further treatment, recurrence, sex, and spread

The mPFS of all patients was 5.8 months (95%CI: 5.3–6.4 months), mOS was 10.3 months (95%CI: 6.8–13.8 months); 1y-PFS was 17.2% (95%CI: 3.7% ~ 30.7%), 1y-OS was 42.9% (95%CI: 23.5% ~ 57.7%) (Table 2, Figure S1). In univariate analysis, PFS and OS were related to recurrence, age, tumor location, and further anti -tumor treatment (Table 2). For PFS, pediatric patients have a worse prognosis than adults (log-rank *P* = 0.001, Table 2, Fig. 4a), tumors out of pons have a better prognosis (log-rank *P* = 0.013, Table 2, Fig. 4a). The results of overall Cox survival analysis showed that age, the best response, and recurrent tumor were independent prognostic factors (Table 2). The mOS of patients with tumor disseminating before treatment was only 6.0 months.

**Table 2:** Univariate prognostic analysis of all patients

Factors	OS			PFS		
	**Median**	**95%CI**	***P*** **value**	**Median**	**95%CI**	***P*** **value**
**Gender**						0.345
Male	9.8	4.09–15.57	0.413	5.8	5.47–6.22	
Female	11.0	7.35–14.71		6.1	3.94–8.28	
**Age**						
≥ 18	**36.7**	11.67–61.66	** < 0.001**	**10.0**	0–29.784	**0.001**
< 18	**8.7**	5.48–11.99		**4.8**	3.108–6.551	
**Course**						
Newly diagnosed	**15.6**	5.62–22.39	**0.041**	**9.0**	5.839–12.231	**0.038**
Recurrent	**7.8**	3.88–11.85		**4.8**	2.168–7.491	
**Location**						
Pons	**9.0**	7.20–13.18	** < 0.001**	**9.3**	3.344–15.251	**0.013**
Out of pons	**30.4**	0–70.74		**5.8**	3.492–8.204	
**Diameter**						
≤ 3	13.1	9.46–16.74	0.926	6.6	3.39–11.22	0.795
> 3	9.5	7.00–12.00		5.8	5.42–6.28	
**WHO Grade**						
2	9.5	8.42–10.57	0.304	5.8	2.25–9.45	0.143
3	6.7	4.43–8.97		4.0	2.19–5.76	
4	14.5	7.92–21.08		7.3	4.81–13.26	
**ATRX**						
Loss	7.9	4.62–11.11	0.075	5.7	2.01–9.42	0.133
Complete	18.1	12.14–24.06		9.0	7.82–14.13	
**Ki67 (%)**			0.353			0.150
≤ 5	19.6	-		12.8	-	
> 5	11.0	5.08–17.00		5.7	3.79–7.90	
**MGMT**			0.939			0.492
Methylated	9.8	2.46–17.21		7.3	2.00–12.58	
Unmethylated	12.1	6.58–17.69		5.8	4.91–7.04	
**Surgical intervention**						
Biopsy only	19.6	-	0.125	12.8	-	0.698
Partial resection	9.0	6.64–11.36		5.8	4.47–7.22	
Gross/sub total	30.4	14.89–45.91		9.1	0–22.81	
**Responses**			**0.023**			** < 0.001**
PR	13.1	7.87–18.33		10.0	5.94–14.10	
MR	12.1	6.69–17.57		5.8	2.79–9.32	
SD	11.0	2.41–19.66		6.1	4.99–7.23	
PD	5.4	2.58–8.26		1.1	0.34–1.76	
**Salvage treatment**						
Anti-tumor	**13.1**	8.94–17.26	**0.002**	N/A		
Supportive	**6.7**	4.50–8.84

For OS, pediatric patients have a worse prognosis than adults (log-rank *P* < 0.001, Fig. 4b), tumors out of pons have a better prognosis (log-rank *P* < 0.001, Table 2, Fig. 4b). The prognosis of patients who just received supportive care after treated in our study is significantly worse than that of patients who continued to receive further anti-tumor therapy(log-rank *P* = 0.002, Table 2, Fig. 4b).

To clarify the efficacy of the combined regimen in newly diagnosed patients (n = 16) and recurrent patients (n = 16), we analyzed the prognosis of the two groups of patients separately. For OS, recurrent patients have the worse prognosis than the whole and newly diagnosed patients (log-rank *P* = 0.041, Table 2, Fig. 4b). For PFS, there is a similar trend (log-rank *P* = 0.038, Table 2, Fig. 4a).

Among newly diagnosed patients (n = 16), the mPFS was 9.0 months (95% CI: 5.8–12.2 months), and the 1y-PFS was 21.1% (95% CI: 5.2%- 51%). The mOS was 15.6 months (95%CI: 7.3–23.8 months), and the 1y-OS 54.5% (95%CI: 29.4%-79.5%) (Table S1). Univariate analysis showed that among newly diagnosed patients (Table S2, Figure S2), the mOS and mPFS of adult and pediatric were 36.7 months vs 9.8 months (*P* = 0.001), 25.9 months vs 6.0 months (*P* = 0.011); the mOS and mPFS were 9.8 months vs 30.4 months (*P* = 0.008) and 6.0 months vs 11.0 months (*P* = 0.029) for patients with tumors located in the pons and outside the pons, respectively.

Among recurrent patients (n = 16), the mPFS was 4.8 months (95% CI: 2.2–7.5 months), 6 m-PFS was 25% (95%CI: 3.8%-46.2%), and the 1y-PFS was 6.3% (95%CI: 0–18.3%) (Table S3). The mOS was 7.8 months (range: 2.1–39.2 + months), and 1y-OS was 18.8% (95%CI: 10.2%-49.5%). Like newly diagnosed patients, univariate analysis showed that OS in recurrent patients was also related to age and tumor location (Table S4, Figure S3). However, there was no significant difference in PFS among recurrent patients with different onset ages and tumor locations but was related to whether the tumor spread (Table S4, Figure S3), which possibly because the proportion of recurrent patients who had disseminated before treatment was relatively high (43.8%), and the extent of the lesion has a greater impact on the curative effect.

### Long-term benefit for patients and follow-up

Among all patients, there were 3 patients who completed the whole treatment cycle and entered the follow-up period. These 3 patients were adults, and the main body of the tumor was in the thalamus without brain stem involvement. Case 7 was a recurrent patient, and case 8 and case 14 were newly diagnosed. The best response evaluation of case 7 was PR, case 14 was SD, case 8 underwent total tumor resections, and no intracranial lesions were measured after surgery. Progression-free survival was 29.0, 25.9 and 27.3 months, and overall survival was 39.8, 30.4 and 36.7 months, respectively. Unfortunately, the three patients developed progression at 6.9, 2.4, and 2.1 months after discontinuation, respectively, and died of tumor progression at 10.3, 4.2, and 9.0 months, respectively, despite receiving other anti-tumor therapies after treatment.

### Toxicity

A total of 236 cycles of 32 patients in treatment were available for safety assessment. Overall, the toxicity of the combination therapy was relatively well tolerated. Table 3 shows the grade 3–4 adverse reactions during the treatment. The most common adverse reaction was myelosuppression. Grade 3 toxicities in all patients were leukopenia (5.08%), neutropenia (4.24%), lymphopenia (2.12%), thrombocytopenia (1.69%), anemia (1.27%), ALT increased (0.42%), AST increased (0.42%), proteinuria (1.69%), decreased appetite (0.42%), vomiting (0.85%), diarrhea (0.42%), hypertension (1.27%), palmar-plantar erythrodysesthesia symptoms (2.12%), leukoencephalopathy (0.42%), memory impairment (0.85%), and depression (0.42%); Grade 4 adverse events included neutropenia (2.12%), thrombocytopenia (2.12%) and proteinuria (1.69%). All the adverse events mentioned above were relieved after symptomatic treatment or dose reduction (dose reduction in 2 cases), and no grade 5 adverse events were observed. While treatment, 2 patients paused apatinib due to toxicities, of which 1 patient paused the application of apatinib due to leukoencephalopathy and memory impairment; the other patient discontinued the apatinib due to grade 3 to 4 proteinuria.

**Table 3:** Adverse Events of Grade 3–4

Adverse Events	Grade 3		Grade 4	
	**Number**	**Percentage (%)**	**Number**	**Percentage (%)**
Neutropenia	10	4.24	5	2.12
Leukopenia	12	5.08	0	0.00
Lymphopenia	5	2.12	0	0.00
Thrombocytopenia	4	1.69	5	2.12
Anemia	3	1.27	0	0.00
ALT increased	1	0.42	0	0.00
AST increased	1	0.42	0	0.00
Proteinuria	3	1.27	4	1.69
Decreased appetite	1	0.42	1	0.42
Vomiting	2	0.85	0	0.00
Diarrhea	1	0.42	0	0.00
Hypertension	3	1.27	0	0.00
Palmar-plantar erythrodysesthesia symptoms	5	2.12	0	0.00
Leukoencephalopathy	1	0.42	0	0.00
Memory impairment	2	0.85	0	0.00
Depression	1	0.42	0	0.00

## Discussion

This report presents the findings of a retrospective study examining the efficacy of temozolomide combined with apatinib in the treatment of diffuse midline gliomas (DMG). As the first cohort report of this treatment combination in the context of DMG, our data suggests that this regimen holds promise in terms of efficacy while maintaining acceptable levels of toxicity. Notably, both apatinib and temozolomide were administered orally, reducing the need for hospitalization. This aspect of the treatment regimen may contribute to improved patient compliance and economic feasibility. The mOS for all enrolled patients included in the studywas 10.3 months, with a 1y-OS of 42.9%. The mPFS was 5.8 months, with a 1y-PFS was 17.2%. Among newly diagnosed patients, the mPFS was 9.0 months, and the 1y-PFS was 21.1%. Furthermore, the mOS for this subgroup was 15.6 months, with a 1y-OS of 54.5%.

The prognosis of patients within this cohort exhibits a significant correlation with age at diagnosis, aligning with findings from other large-scale cohort studies [5, 20]. Adults demonstrate a notably prolonged mOS of 36.7 months, in stark contrast to the 8.7 months observed for pediatric patients. Additionally, survival outcomes are influenced by tumor location and the presence of metastasis. Patients with tumors located in the pons demonstrate a markedly reduced mOS of 9.0 months, whereas those with tumors situated outside the pons experience a more favorable mOS of 30.4 months. Notably, patients whose tumors disseminated before treatment experienced a markedly reduced mOS of only 6.0 months. Furthermore, the decision to continue anti-tumor therapy emerges as an important factor affecting prognosis. In this cohort, patients who discontinued anti-tumor therapy post-treatment at our facility displayed a mOS of 6.7 months, while those who continued with such therapy displayed a notably prolonged mOS of 13.1 months. Contrary to findings from prior case cohort studies, no survival advantage was evident due to specific molecular pathological changes, such as ATRX loss and H3.1 mutation, such as ATRX loss and H3.1 mutation [5, 20]. This discrepancy may be attributed to the limited number of patients displaying the aforementioned molecular alterations.

One noteworthy observation is that the median OS in adult patients is 36.7 months in this series, higher than in two other studies: 27.6 months in Schulte et al. [20] and 16.0 months in Zheng et al. [5]. Consequently, the novel therapy investigated in our study warrants further investigation in adult DMG patients.

### Effect on newly diagnosed patients

This study indicates that the combined regimen offers improved efficacy compared to temozolomide monotherapy for newly diagnosed DMG patients. Table S5 compares the efficacy and patient characteristics between the combined regimen and previous monotherapy approaches. In the ACNS0126 trial [21], 63 pediatric patients with DIPG received temozolomide monotherapy, resulting in a mPFS of 6.1 months, and a mOS of 9.6 months; findings from the CNS200704 trial also found similar results [22]. Aihara et al. [23] treated 10 adult patients with thalamic tumors using temozolomide or ACNU, yielding an mPFS of 6.0 months and an mOS of 10.4 months, aligning with the data reported by Jang et al. [24]. According to Schulte's research [20], 90% of 50 adult patients received systemic therapy with temozolomide monotherapy during or after radiotherapy. The mPFS for this cohort was 9.6 months, with a mOS of 29.6 months. However, patients included in this study were adults, and over 90% of the tumors were situated outside the pons. This demographic and tumor distribution could contribute to a notably improved prognosis compared to other cohorts. Furthermore, combined treatment exhibited superior efficacy compared to the limited monotherapy available. These findings suggest that apatinib may enhance the effectiveness of temozolomide in combination regimens, which is consistent with the findings of several preclinical studies [9, 10]. These studies have demonstrated that the inhibition of VEGFR-2 could enhance the efficacy of temozolomide. Downregulation of VEGFR-2 led to reduced cell proliferation and increased sensitivity of glioma cells to temozolomide-induced G2 cell cycle arrest.

It is noteworthy that the mOS reported in primary adult patients by Schulte et al. [20] was 27.6 months, with 20 out of 43 patients in the series receiving treatment with bevacizumab. Interestingly, three patients within the corresponding cohort of our study, (Patient ID: 5, 16, and 28), who were treated with bevacizumab, showed a seemingly prolonged survival (Table S1). It should be noted that bevacizumab appears to have a more favorable effect than other supportive care options on tumors that have progressed after treatment with TMZ plus apatinib. However, large cohorts and mechanistic studies are required to validate these findings.

#### Effect on recurrent patients

Previous studies focusing on patients with recurrent DMG are limited. Table S6 compares the efficacy and patient characteristics between the combination regimen and previous cohorts. Our data reveals that the 7 out of 16 recurring patients exhibited disseminated lesions, suggesting that the tumor dissemination is a frequent occurrence in the later stages of the disease. Consequently, a comprehensive whole-brain and spinal cord magnetic resonance examination is necessary. In terms of PFS data, our protocol (4.8 months) likely outperforms most existing studies and is comparable to the results observed in supratentorial recurrent GBM (4.2 months, EORTC26101). However, our results do not surpass those of ONC201 (OS 21.7 months, PFS 7.3 months), potentially due to differing mechanisms of action between these drugs. Nevertheless, there was no significant difference observed in the survival of patients with recurrence [25].

The results indicate that the efficacy of combination therapy was similar to that of re-radiation therapy [26]. When compared with nimotuzumab alone, the efficacy of combination therapy may be superior [27], although no significant difference was observed in the efficacy of other combination therapies [28, 29]. Tumors that have undergone systemic treatment tend to develop resistance to anti-tumor therapy, and patients' overall health deteriorates after multiple lines of therapy, making them less tolerant to high-dose treatments [14]. Consequently, recurrent tumors pose greater challenges in terms of treatment.

In the treatment of diffuse midline glioma, the mode of administration must be carefully considered. Oral administration of apatinib in combination with temozolomide offers the advantages of convenience, simplicity, and ease of management, as validated by our clinical practice. On the other hand, for patients with severe brain stem damage and swallowing dysfunction, oral medication poses challenges. Therefore, alternative approaches and regimens need to be explored for these patients.

From our study, involving a limited sample of 3 out of 16 patients with long-term survival of diffuse midline glioma, we found that adults, patients with tumors located in the thalamus may derive benefit from our treatment strategy. These patients could represent a clinically sensitive subgroup responsive to this treatment regimen. However, it is important to note that patients who respond effectively to this treatment often experience recurrence within six months, highlighting the importance of long-term anti-tumor therapy.

Our study has several limitations. It was a single group study that did not include a control group. The single-center nature of our study introduces a potential for selection bias, and the absence of molecular data further limits our ability to identify biomarkers associated with treatment efficacy. We believe that future studies could benefit from improved study designs statistical analyses. Furthermore, apatinib remains an experimental drug in the context of neurotumors, which may impose financial burdens on families and affect the continuity of patient treatment.

## Conclusion

The present study provides preliminary evidence suggesting that combination therapy utilizing apatinib and temozolomide demonstrates favorable efficacy and may outperform temozolomide monotherapy, particularly in newly diagnosed adults with tumors located outside the pons. This finding warrants further investigation in adult DMG patients. Additionally, the manageable toxicity profile, coupled with the convenience of oral administration eliminates the need for hospitalization.

### Supplementary Information

Supplementary Material 1.

## Data Availability

All analyzed data are included in this published article. The original data are available upon reasonable request to the corresponding author.
