# Correction: Apatinib combined with temozolomide in diffuse midline glioma: a novel and effective therapy

**DOI:** 10.1186/s12885-024-12600-3

**Published:** 2024-07-11

**Authors:** Yu-An Li, Chuan Zhao, Jing-Jing Ge, Cheng Li, Feng-Jun Xue, Shao-Pei Qi, Chi Zhao, Chen-Chen Kong, Jun-Ping Zhang

**Affiliations:** Department of Neuro-Oncology, Sanbo Brain Hospital, Capital Medical University, Beijing, China

**Correction**: ***BMC Cancer*** 24, **754 (2024)**

10.1186/s12885-024-12373-9

Following publication of the original article [1], a typesetting error was reported. The equal contribution statement was missing in the published article. YuAn Li and Chuan Zhao are co-first authors.

The original article [1] has been corrected.
