# Supplementary material for: Apatinib combined with temozolomide in diffuse midline glioma: a novel and effective therapy
Source: BMC Cancer. 2024 Jun 21;24:754. doi: 10.1186/s12885-024-12373-9 (PMC11193221; doi:10.1186/s12885-024-12373-9)
Supplement: Supplementary file 1 — Supplementary Material 1. [file 12885_2024_12373_MOESM1_ESM.docx]

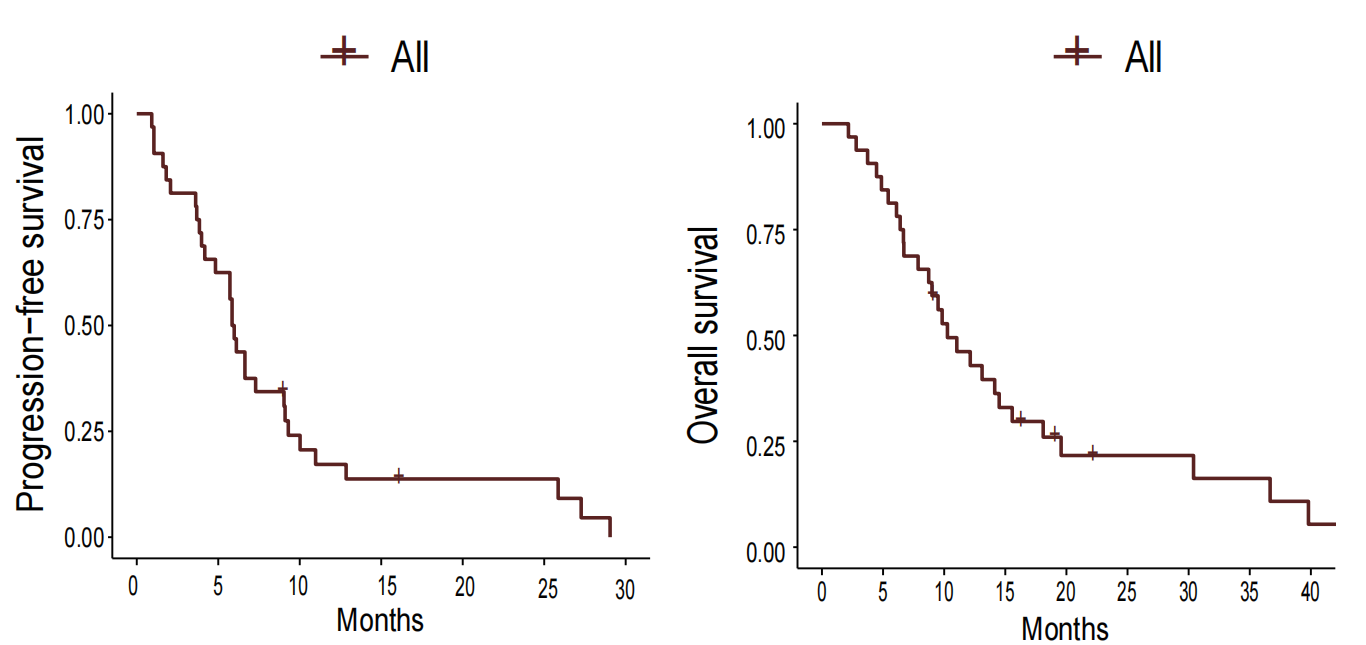


**Figure S1. Progression-free survival and overall survival curves for all patients**


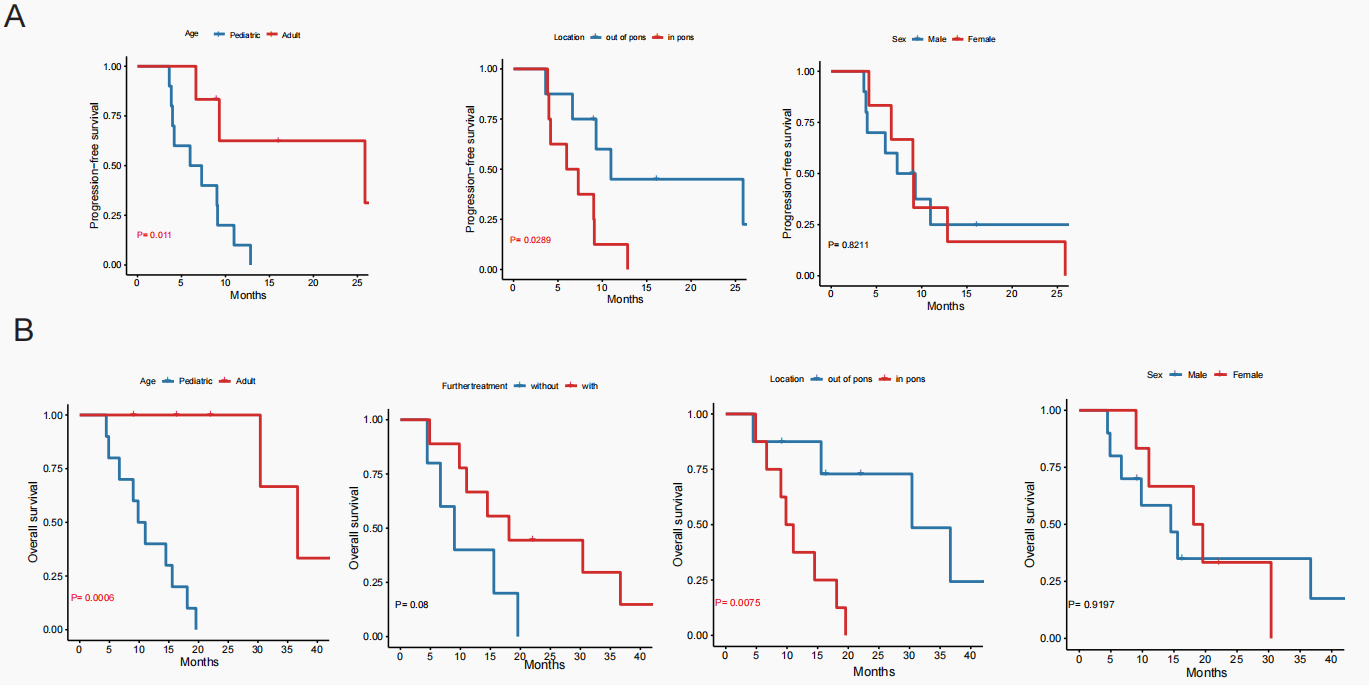


**Figure S2.** **Prognostic analysis of newly diagnosed patients**

**A** Progression-free survival was compared between groups based on age, tumor location, sex.

**B** Overall survival was compared between groups based on age, tumor location, further treatment, sex.


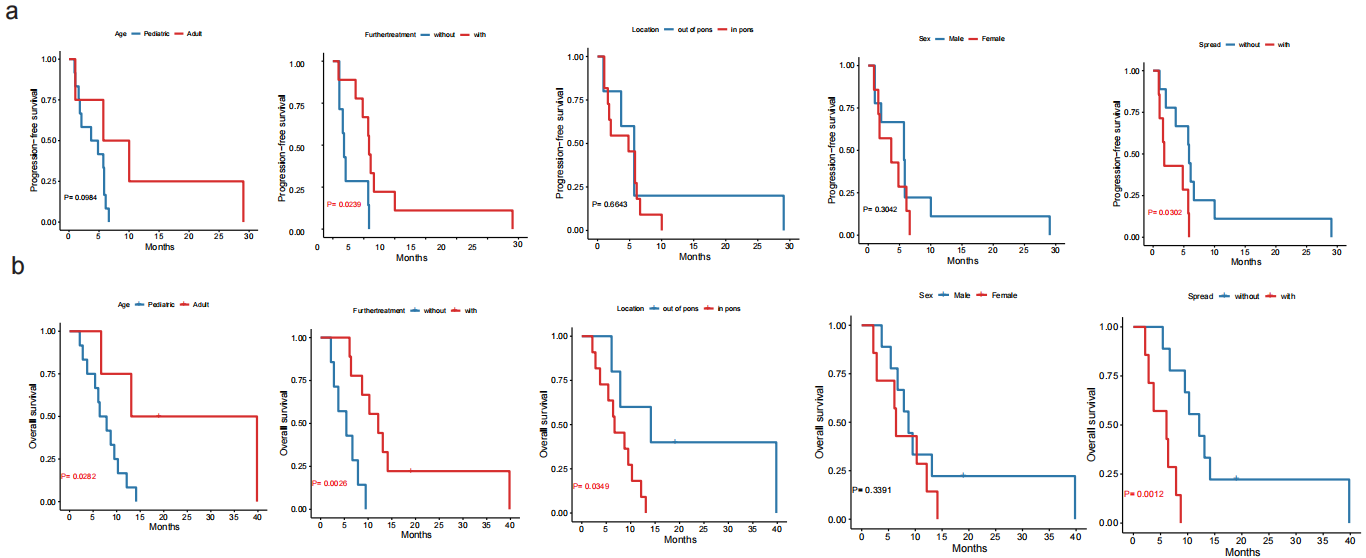


**Figure S3. Prognostic analysis of recurrent patients**

**A** Progression-free survivals were compared between groups based on age, tumor location, further treatment, sex, and spread.

**B** Overall survivals were compared between groups based on age, tumor location, further treatment, sex, and spread.


**TableS1. Treatment in newly diagnosed patients**

| **Patient ID** | **Gender** | **Age** | **Location** | **Treament history** | **Progressed** | **PFS(months)** | **Salvage treatment** | **Status** | **OS(months)** |
| --- | --- | --- | --- | --- | --- | --- | --- | --- | --- |
| 3 | M | 9 | Pon | Partial+XRT | Yes | 6.0 | Supportive care | Dead | 6.7 |
| 5 | M | 31 | Thalamus | Subtotal+XRT/TMZ | Yes | 9.3 | Bev mono-/combined therapy | Alive | 42.6+ |
| 6 | M | 10 | Pon | Partial+XRT/TMZ | Yes | 4.0 | Clinical trials | Dead | 4.9 |
| 8 | F | 23 | Thalamus | Total+XRT/TMZ | Yes | 25.9 | Reoperation/Clinical trials | Dead | 30.4 |
| 9 | F | 8 | Pon | Partial+XRT/TMZ | Yes | 4.2 | Supportive care | Dead | 9.0 |
| 11 | M | 5 | Pon | Partial+XRT | Yes | 7.3 | Bev mono-/combined therapy | Dead | 9.8 |
| 12 | M | 9 | Thalamus | Total+XRT/TMZ | Yes | 3.6 | Supportive care | Dead | 4.5 |
| 13 | M | 5 | Pon | Partial+XRT/TMZ | Yes | 3.8 | Reoperation/Clinical trials | Dead | 14.5 |
| 14 | F | 15 | Pon | Subtotal+XRT/TMZ | Yes | 9.1 | Clinical trials | Dead | 18.1 |
| 16 | M | 53 | Thalamus | Partial+XRT/TMZ | Yes | 27.3 | Reoperation/Bev mono-/combined therapy | Dead | 36.7 |
| 18 | M | 15 | Ventricles | Partial+XRT/TMZ | Yes | 11.0 | Supportive care | Dead | 15.6 |
| 22 | F | 12 | Pon | Biopsy+XRT/TMZ | Yes | 12.8 | Supportive care | Dead | 19.6 |
| 25 | M | 47 | Thalamus | Subtotal+XRT/TMZ | Not available | 9.1+ | Not available | Not available | 9.1+ |
| 26 | M | 35 | Spine | Partial+XRT/TMZ | Not available | 16.3+ | Not available | Not available | 16.3+ |
| 28 | F | 55 | Ventricle | Partial+XRT/TMZ | Yes | 6.6 | Bev mono-/combined therapy | Alive | 22.2+ |
| 32 | F | 7 | Pon | Partial+XRT/TMZ | Yes | 9.0 | Bev mono-/combined therapy | Dead | 11.0 |

XRT radiation therapy; XRT/TMZ, Temozolomide concurrent chemoradiotherapy; Bev Bevacizumab

**Table S2. Univariate prognostic analysis of newly diagnosed patients**

| **Factors** | **OS** | | | **PFS** | | |
| --- | --- | --- | --- | --- | --- | --- |
|  | **median** | **95%CI** | **P Value** | **median** | **95%CI** | ***P* Value** |
| **Gender**  male  female | 14.5  18.1 | 6.52-22.48  7.86-28.35 | 0.920 | 7.3  9.0 | 2.72-11.86  6.08-11.99 | 0.821 |
| **Age** |  |  |  |  |  |  |
| ≥18 | **36.7** | 26.64-46.70 | **0.001** | **25.9** | 0.370-51.341 | **0.011** |
| ＜18 | **9.8** | 6.68-12.98 |  | **6.0** | 1.143-10.816 |  |
| **Location** |  |  |  |  |  |  |
| Pons | **9.8** | 7.02-12.65 | **0.008** | **6.0** | 1.654-10.305 | **0.029** |
| Out of pons | **30.4** | 11.10-49.70 |  | **11.0** | 6.852-15.095 |  |
| **Diameter** |  |  | 0.636 |  |  | 0.914 |
| ≤3 | 9.8 | - |  | 7.3 | - |  |
| >3 | 15.6 | 9.47-21.66 |  | 9.0 | 4.52-13.55 |  |
| **WHO Grade** |  |  | 0.806 |  |  | 0.855 |
| 2 | 9.0 | - |  | 4.2 | - |  |
| 3 | 4.9 | - |  | 4.0 | - |  |
| 4 | 15.6 | 9.84-21.30 |  | 9.0 | 6.34-11.73 |  |
| **Ki67（%）** |  |  | 0.667 |  |  | 0.264 |
| ≤ 5 | 19.6 | - |  | 12.8 | - |  |
| >5 | 15.6 | 8.89-22.25 |  | 9.0 | 4.40-13.66 |  |
| **MGMT** |  |  | 0.600 |  |  | 0.233 |
| methylated | 30.4 | - |  | 25.9 | - |  |
| unmethylated | 15.57 | 8.23-22.91 |  | 9.0 | 3.96-14.11 |  |
| **Surgical intervention** |  |  | 0.617 |  |  | 0.880 |
| biopsy only | 19.6 | - |  | 12.8 | - |  |
| partial resection | 11.0 | 3.80-18.26 |  | 6.6 | 4.60-8.67 |  |
| Gross/sub total | 30.4 | 8.89-51.92 |  | 9.3 | 4.58-14.01 |  |
| **Responses** |  |  | 0.771 |  |  | **0.043** |
| **PR** | 9.8 | - |  | 7.3 | - |  |
| **SD** | 18.1 | 6.35-29.85 |  | 9.1 | 5.98-12.22 |  |
| **PD** | 14.5 | - |  | 3.8 | - |  |
| **Salvage treatment** |  |  |  | N/A | | |
| Anti-tumor | 18.1 | 7.58-28.61 | 0.080 |  |  |  |
| Supportive | 9.0 | 3.99-14.01 |  |  |  |  |

**Table S3. Treatment in recurrent patients**

| Patient ID | Gender | Age(years) | **Location** | Spread | Times of recurrence | Initial treatment | First PFS(months) | Second-line treatment | Second PFS(months) | **Current Treatment** | PFS(months) | **Status** | **OS**(months) |
| --- | --- | --- | --- | --- | --- | --- | --- | --- | --- | --- | --- | --- | --- |
| 1 | M | 13 | Pon | No | 1 | Biopsy+XRT/TMZ+adjTMZ | 10.8 | - | - | Partial+TMZ/Apa | 2.1 | Dead | 5.4 |
| 2 | F | 8 | Pon | Yes | 1 | Partial+XRT/TMZ+ Cytotoxic drugs | 5.7 | - | - | TMZ/Apa | 1.8 | Dead | 2.8 |
| 4 | M | 13 | Pon | No | 1 | Partial+XRT/TMZ+ Cytotoxic drugs | 15.2 | - | - | TMZ/Apa | 5.8 | Dead | 9.4 |
| 7 | M | 31 | Thalamus | No | 1 | Partial+XRT/TMZ+adjTMZ | 5.5 | - | - | TMZ/Apa | 29.0 | Dead | 39.2 |
| 10 | M | 28 | Pon | No | 1 | Partial+XRT/TMZ+adjTMZ | 5.4 | - | - | TMZ/Apa | 10.0 | Dead | 12.9 |
| 15 | M | 17 | Thalamus | Yes | 1 | Partial+XRT/TMZ+adjTMZ | 6.1 | - | - | TMZ/Apa | 1.1 | Dead | 3.7 |
| 17 | F | 7 | Pon | No | 1 | Partial+XRT/TMZ/Bev+adjTMZ | 9.8 | - | - | TMZ/Apa | 6.1 | Dead | 10.2 |
| 19 | M | 26 | Pon | No | 1 | Partial+XRT | 23.7 | - | - | TMZ/Apa | 1.1 | Dead | 6.6 |
| 20 | F | 9 | Pon | Yes | 1 | Partial+XRT/TMZ+adjTMZ | 20.6 | - | - | TMZ/Apa | 0.9 | Dead | 6.0 |
| 21 | M | 33 | brachium pontis | No | 2 | Total+XRT/TMZ+adjTMZ | 33.9 | reXRT/Nimot+adjTMZ | 15.6 | TMZ/Apa | 5.7 | Alive | 18.8+ |
| 23 | F | 8 | Pon | Yes | 1 | Partial+XRT/TMZ | 3.2 | - | - | TMZ/Apa | 1.6 | Dead | 2.1 |
| 24 | F | 8 | Pon | Yes | 2 | Partial | 2.4 | XRT/TMZ | 2.9 | TMZ/Apa | 4.8 | Dead | 6.3 |
| 27 | F | 10 | Thalamus | No | 1 | Subotal+XRT/TMZ+adjTMZ | 6.0 | - | - | TMZ/Apa | 3.7 | Dead | 13.9 |
| 29 | F | 10 | Pon | No | 1 | Partial+XRT/TMZ+adjTMZ | 3.4 | - | - | TMZ/Apa | 6.6 | Dead | 12.0 |
| 30 | M | 13 | Thalamus | Yes | 1 | Partial+XRT/TMZ+adjTMZ | 6.9 | - | - | TMZ/Apa | 5.8 | Dead | 8.6 |
| 31 | M | 10 | Ventricle | Yes | 1 | Partial+XRT/TMZ | 4.5 | - | - | TMZ/Apa | 5.7 | Dead | 7.8 |

**Table S4. Prognostic analysis of recurrent patients**

| **Factors** | **OS** | | | **PFS** | | |
| --- | --- | --- | --- | --- | --- | --- |
|  | **median** | **95%CI** | **P Value** | **median** | **95%CI** | **P Value** |
| **Gender**  male  female | 8.6  6.3 | 6.10-11.12  5.54-7.08 | 0.339 | 5.7  3.7 | 0.39-11.04  0-8.49 | 0.304 |
| **Age** |  |  |  |  |  |  |
| ≥18 | **12.9** | 0-34.228 | **0.028** | 5.7 | 0-8.364 | 0.098 |
| ＜18 | **6.3** | 3.357-9.263 |  | 3.7 | 0-14.506 |  |
| **Location** |  |  |  |  |  |  |
| Pons | **6.6** | 3.158-10.042 | **0.035** | 4.8 | 3.931-7.502 | 0.664 |
| Out of pons | **13.9** | 0.661-27.199 |  | 5.7 | 1.559-8.100 |  |
| **Spread or not** |  |  |  |  |  |  |
| Spread | **6.0** | 6.788-17.132 | **0.001** | **1.8** | 1.301-2.313 | **0.030** |
| no | **12.0** | 0.031-11.989 |  | **5.8** | 5.464-6.232 |  |
| **MGMT** |  |  | 0.114 |  |  | 0.109 |
| methylated | 6.0 | - |  | 0.9 | - |  |
| unmethylated | 8.6 | 5.88-11.34 |  | 5.7 | 2.31-9.13 |  |
| **Responses** |  |  | **0.007** |  |  | **<0.001** |
| PR | 12.9 | - |  | 10.0 | - |  |
| MR | 12.0 | 6.60-17.32 |  | 5.8 | 2.38-9.32 |  |
| SD | 7.8 | 4.09-11.41 |  | 5.7 | 4.71-6.72 |  |
| PD | 5.4 | 1.68-9.16 |  | 1.1 | 0.91-1.19 |  |
| **Salvage treatment** |  |  |  | N/A | | |
| Anti-tumor | **12.0** | 6.788-17.132 | **0.003** |  |  |  |
| Supportive | **5.4** | 0.955-9.885 |  |  |  |  |

**Table S5. Comparison of efficiency in newly diagnosed patients with history**

| **Year** | Author | Regimen | Number of patients | The proportion of non-pontine patients | The proportion of adult patients | **mPFS(months)** | **mOS(months)** |
| --- | --- | --- | --- | --- | --- | --- | --- |
| Current | Yu-an Li | XRT or XRT/TMZ+adjTMZ/APA | 16 | 50.0% | 37.5% | 9.0 | 15.6 |
| 2011 | Cohen KJ | XRT/TMZ+adjTMZ | 58 | 0 | 0 | 6.1 | 9.6 |
| 2013 | Bailey S | XRT/TMZ+adjTMZ | 43 | 0 | 14.0% | 5.6 | 9.5 |
| 2014 | Aihara K | XRT/TMZ or ACNU | 10 | 100% | 90.0% | 6.0 | 10.4 |
| 2020 | Schulte JD | XRT or XRT/TMZ±adjTMZ | 60 | 91.7% | 100% | 9.6 | 29.6 |
| 2022 | Jang SW | XRT or XRT/TMZ±adjTMZ | 24 | 66.4% | 54.2% | 3.9 | 10.4 |

**Table S6. Comparison of efficiency in recurrent patients with history**

| Year | Author | Regimen | Number of patients | Proportion of disseminated patients | Proportion of non-pontine patients | Proportion of adult patients | **mPFS**  **(months)** | **1-year PFS rate** |
| --- | --- | --- | --- | --- | --- | --- | --- | --- |
| Current | Yu-an Li | TMZ/APA | 16 | 43.8% | 37.5% | 25.0% | 4.8 | 6.3% |
| 2017 | Lassaletta A | reXRT | 16 | 12.5% | 0 | 0 | 4.0 | 6.3% |
| 2018 | Broniscer A | Dasatinib/Crizotinib | 25 | 12.0% | 56.0% | 8.0% | 1.4 | - |
| 2014 | Bartels U | Nimotuzumab | 44 | - | 0 | 0 | 1.7 | 2.3% |
| 2021 | EI-Khouly | CPT11+Bev+ Erlotinib | 9 | - | 0 | 0 | 3.2 | 11.1% |
